# Identification of Antigenic and Immunogenic Proteins of Toxoplasma gondii in Human and Sheep by Immunoproteomics

**Published:** 2018

**Authors:** Mohammad Taghi AHADY, Nasser HOGHOOGHI-RAD, Rasool MADANI, Ahmad Reza ESMAEILI RASTAGHI

**Affiliations:** 1. Dept. of Microbiology, Science and Research Branch, Islamic Azad University, Tehran, Iran; 2. Dept. of Parasitology, Pasteur Institute of Iran, Tehran, Iran

**Keywords:** *Toxoplasma gondii*, Antigens, Immunoproteomics, Enolase, Rhoptry’s proteins, GRA14

## Abstract

**Background:**

Toxoplasmosis is a parasitic disease caused by the intracellular protozoan parasite, *Toxoplasma gondii*, which can infect humans and warm-blooded animals. This infection can lead to still birth and abortion among some susceptible hosts especially sheep and human in pregnancy. Development of a vaccine against *T. gondii* infection is very important-especially for use in immunocompromised patients, pregnant women, and sheep. Different antigens of *T. gondii* can be potential candidates for immunization. The aims of this study were to identify the immunodominant and antigenic proteins of *T. gondii* in sheep and man.

**Methods:**

Tachyzoites’ proteins were separated by two-dimensional polyacrylamide gel electrophoresis (2-DE), and subjected to western blot analysis probed with *T. gondii* positive sera of sheep and human (Biotechnology Department of Pasteur Institute of Tehran, Iran, from April 2016 to March 2017). Finally, the immunoreactive proteins were identified by mass spectrometry (MALDI-TOF/MS and MS/MS) technique.

**Results:**

Five immunoreactive and antigenic proteins were recognized by *Toxoplasma* positive sera of human and sheep. These identified proteins were Enolase 2, rhoptry protein 4 (ROP4), dense granular protein 14 (GRA14), rhoptry protein 15 (ROP15) and rhoptry protein 9 (ROP9).

**Conclusion:**

The identified immunodominant proteins have potential to be used as diagnostic antigens and as diagnostic markers of *Toxoplasma* infection in sheep and human.

## Introduction

Toxoplasmosis is a worldwide infection caused by *Toxoplasma gondii*, an obligatory intracellular parasite. This protozoan infects virtually all warm-blooded animals including human and livestock. *T. gondii* has been recognized as one of the main causes of abortion in human and sheep. It can also lead to death in immune-deficient patients. Cattle and horses are considered highly resistant to clinical toxoplasmosis (1).

Despite several available diagnostic techniques, we need accurately, reliable and non-invasive methods for easily determining the acute/chronic infections of *T. gondii* (2). On the other hand, development of a vaccine against *T. gondii* infection is very important-especially for use in immunocompromised patients, pregnant women and sheep (3). Different antigens of *T. gondii* can be potential candidates for immunization. Excretory-Secretory Antigens (ESA) have important roles in induction of immune system responses. Dense granules, micronemes, and rhoptries are secretory organelles in this protozoan (4).

The immunoprotective value of rhoptry protein5 (ROP5) was studied in BALB/c mice using a recombinant form of the protein alone and in combination with rSAG1. rROP5 could induce significant cellular and humoral immune responses (5). Antigenicity of soluble tachyzoite antigen (STAg) should be investigated in order to find out protective antigens for diagnosis of and for immunization against toxoplasmosis. Immunoproteomic method was carried out for identifying the proteins of tachyzoites (6). In this study, nine novel types of immunogenic proteins were obtained that might be potential candidates for a vaccine development for toxoplasmosis (6).

A group of researchers in China succeeded to identify 18 immunoreactive proteins of *T. gondii* using 2-D immunoblotting technique and MALDI-TOF MS, and MS/MS analyses. Among these identified proteins, actin, catalase, GAPDH, and three hypothetical proteins were indicated to have potential as diagnostic marker for toxoplasmosis (7). Mexican researchers studied the proteins of the subpellicular cytoskeleton of *T. gondii* and reported 95 proteins (8). IgM and IgG were present in allantoic and amniotic fluids of *T. gondii* infected pregnant ewes. They also detected two groups of antigens, one with ∼ 22 kDaAa and the other of ∼ 30 kDaa, by two-dimensional immunoblots (9).

Dense granular (GRA) proteins of *T. gondii* are the most important diagnostic markers for detection of toxoplasmosis by serological assay. In Iran, the GRA7 for the production of DNA vaccine was introduced against toxoplasmosis (10). The importance of rGRA7 was in the diagnosis of toxoplasmosis in people suffering from cancer (11). Dziadek et al. assessed the vaccine potential of some proteins of rhoptry and dense granules including rROP2 and rGRA4 (12).

Serological assays are the most common tests for toxoplasmosis diagnosis. Specificity and sensitivity of these methods depend on diagnostic antigens used in the assays. In order to improve the quality of these methods, researchers are going to recommend some recombinant antigens for the serodiagnosis of acute and chronic toxoplasmosis. The recombinant SAG1 protein could be an alternative marker for detection of acute toxoplasmosis infection (13, 14). The “biochemical and biophysical characterization of recombinant soluble dense granule proteins GRA2 and GRA6” was identified (15).

Many rhoptry proteins have been showed to be key players in *T. gondii* invasion and virulence. Rhoptry neck proteins (RONs) including RONs 2, 4, 5 and 8 have been invasion (16, 17); ROP16 activates the transcription factors STAT3 and STAT6 (18,19). At the time, RON12, ROP47, and ROP48 are not implicated in *Toxoplasma* virulence in mice (20). GRA4, GRA7, MIC6, ROP1, GRA2 and HSP90 antigens were identified (21). The recombination of GRA7, SAG1, and GRA8 antigens were proposed as recombinant proteins for use in IgG ELISA instead of *Toxoplasma* lysate antigens (TLAs) (22). Chimeric *T. gondii* antigen, P35-MAG1 may be more useful than MIC1-ROP1 and MAG1-ROP1 in the preliminary detection of acute toxoplasmosis in humans (23). SAG1, GRA1 and GRA7 recombinant proteins with sensitivity of 100% and 91.1% for diagnosing the acute toxoplasmosis and the chronic infection respectively were proposed instead of TLAs in IgG ELISA test. GRA8, SAG2 and GRA6 recombinant proteins with 88.9% sensitivity and 100% specificity for diagnosis of chronic phase of toxoplasmosis were introduced (24). In 2008, MIC1ex2, MAG1 and MIC3 with 94.4% sensitivity and 100% specificity and in 2010, three recombinant proteins including GRA2, SAG1 and GRA5 with 93.1% sensitivity and 100% specificity, also three other recombinant proteins (ROP1, SAG1 and GRA5) with 94.2% sensitivity and 100% specificity were proposed to be used in IgG ELISA technique (25,26). Enolase2 could play important roles in metabolism, immunogenicity, and pathogenicity of *T. gondii* and that it may serve as a novel drug target and candidate vaccine against *Toxoplasma* infection (27).

This study aimed to determine the immunoreactive and antigenic proteins of *T. gondii.* These proteins should be capable to detect the different types of toxoplasmosis, not only in human but in sheep, too.

## Materials and Methods

### Growth of Toxoplasma gondii in vivo and isolation of tachyzoites

Female BALB/c mice were used to prepare soluble tachyzoites. Tachyzoites of the virulent RH strain (provided by Parasitology Department of Pasteur Institute of Tehran, Iran, from January 2016 to April 2016) were inoculated intraperitoneally and were maintained by intraperitoneal passage in the mice. Parasites were harvested by collecting peritoneal fluid 3 d after infection. The obtained parasites were washed three times with PBS (pH 7.4) by centrifugation (Chilspin, UK) at 4000g for 10 min at room temperature. Then we added 0.20% trypsin solution in order to lyse any other cells and purification of tachyzoites. *T. gondii* tachyzoites were washed with PBS and were centrifuged at 4000g for 10 min. 5.15×10^8^ tachyzoites per ml were counted using a hemocytometer. The supernatant containing purified tachyzoites was harvested and stored at −20 °C until further use.

The study was approved by Ethics Committee of Islamic Azad University, Tehran, Iran.

### Preparation of total proteins from T. gondii

Frozen tachyzoites were disturbed in lysis buffer consisting of 7 M urea, 4% (W/V) CHAPS, 1 mMPhenylmethane sulfonyl fluoride (PMSF), 1% (W/V) DTT, 2 M Thiourea and 0.5% (V/V) immobilized pH gradient buffer for 30 min, then followed by sonication on ice using a sonicator (Hielscher, Germany) for 5 min. Then disrupted tachyzoites were centrifuged by microcentrifuge (BECKMAN COULTER, USA) at 12000g for 10 min, and then the supernatant was obtained. Finally, the protein concentration was determined using the Bradford method (Biophotometr, Eppendorf) (20, 22, 30).

### Collection and preparation of serum samples, and detecting anti-T. gondii IgG

Dye test and IFA assays were used to detect anti-*T. gondii* IgG in sera collected from humans and sheep. Ten positive serum samples of human and 10 positive serum samples of sheep were obtained by dye test and IFA assays. The samples were stored at −20 °C until use. Dye test was used as the gold-standard method. The method involves the staining of *T. gondii* cells with methylene blue, *Toxoplasma* cells become rounded and the nucleus and cytoplasm are deeply stained (1).

### One-dimensional polyacrylamide gel electrophoresis (1-DE) and western blotting

Thirty *μl* of tachyzoite proteins in lysis buffer were mixed with 6 *μl* (ratio 1:5) of loading buffer (Thermo Scientific 26610). Proteins were separated by electrophoresis in 12% polyacrylamide gel, and visualized by staining with Coomassie brilliant blue. Size-separated proteins were electro-transferred from the unstained gel onto a nitrocellulose membrane. The immunological features of these proteins were determined by Coligan protocol and procedure (7, 28).

The membranes were blocked with blocking buffer (Tris-buffered saline or TBS) containing primary antibody for 2 h, washed three times, with TBST, and incubated with secondary antibody (goat anti-human IgG antibody, Bethyl, USA) for 1 h. After the final washing, immunoreactive proteins were visualized by Kodak image station (400 MM PRO) (7).

### Two-dimensional polyacrylamide gel electrophoresis Isoelectric focusing electrophoresis (IEF)

To separate the proteins in the first dimension by isoelectric focusing (IEF), each sample was applied to a ReadyStrip pH 3 to 10 immobilized pH gradient (IPG) strip and allowed to incubate at room temperature overnight. Strips were then placed in the PROTEAN IEF cell apparatus (Bio-Rad, USA). Tachyzoite protein samples were mixed with rehydration buffer containing 8 M urea, 2 M thiourea, 2% CHAPS, 65 mM DDT, 0.2% Bio-lyte, and 0.001% bromphenol blue. After rehydration, IEF was performed at a constant temperature of 20 °C at 250 V for 1 h, 500 V for 1 h, 1000 V for 1 h, and 8000 V for 4 h, for a total of 14000 V. The IPG strips were equilibrated in sodium dodecyl sulfate (SDS) equilibration buffer containing 50 mMTris-Hcl, 6 M urea, 30% W/V glycerol, 65 mM DTT, 2% W/V SDS, and 10 mg/ml dithiothreitol for 15 min. Then the strips were soaked in an alkylation buffer (6 M urea, 87% V/V glycerol, 2% W/V SDS, 75 mMiodoacetamide, 0.04% bromphenol blue, and 1.5 M Tris-Hcl, pH 8.8) for 15 min. The strips were then transferred onto 12% acrylamide gels and subjected to SDS polyacrylamide gel electrophoresis as the second dimension. Strip gels were stained with Coomassie brilliant blue (15, 35).

### Western blotting

The 2-D gels were transferred onto polyvinylidene fluoride (PVDF) membrane (Millipore, USA). The blotted membranes were incubated with *T. gondii*-positive human and sheep sera for 2 h. After 3 washes in TTBS for 5 min, the membranes were incubated with goat anti-human IgG antibody conjugated with horseradish peroxidase (Bethyl, USA) for 1h. The membranes were washed 3 times with TTBS solution for 10 min and once washed with TBS for 5 min. The proteins on these PVDF membranes were visualized with super Enhanced Chemiluminescent Substrate (ECL) (6, 9).

### Identification of protein spots by MS and bioinformatics analysis

After picking up the interest spots by Ettan spot picker and designing spot picking by Decyder software, the gel spots were washed and digested in-gel with modified porcine trypsin, and then spotted on the MALDI plate. MALDI-TOF and TOF/TOF tandem MS/MS were performed on an ABI 4800 Mass Spectrometer (Ultrashield Plus, BRUKER, USA). The MS and MS/MS data were matched to GPS Explorer workstation equipped with MASCOT software (www.matrixscience.com), and the NCBI nr database (ftp://ftp.ncbi.nih.gov/blast/db/FAST/nr.gz), and TOXODB (www.toxodb.org/toxo/) (7).

## Results

**Fig. 1:**
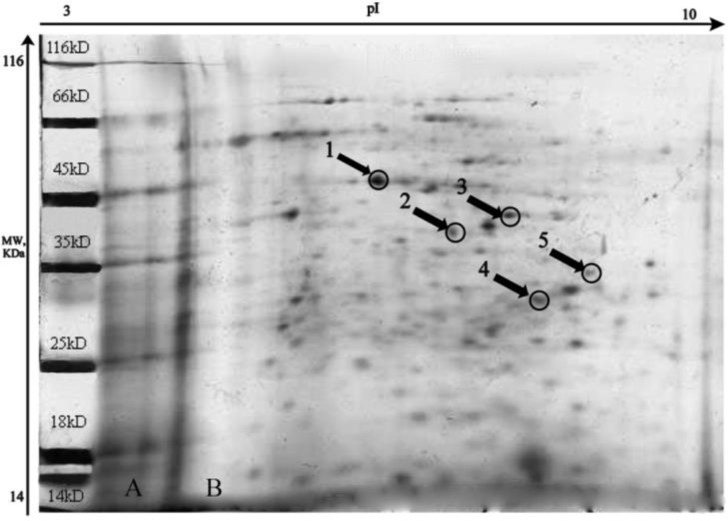
1-D gel electrophoresis of *T. gondii* tachyzoite proteins (A) and 2-D map of the tachyzoite proteome of immunoreactive proteins (B)

In order to identify potential immunogen proteins of *T. gondii*, tachyzoites’ proteins were separated with 2-DE and subjected to western blot analysis probed with *T. gondii* positive human and sheep sera. Five immunoreactive proteins were detected and identified by MALDI-TOF MS and MS/MS analysis (Fig.1). The spots 1, 2 (pertained to sheep serum), 3, 4, and 5 (belonged to human serum) were identified as enolase2, rhoptry protein 4 (ROP4), dense granular protein 14 (GRA14), rhoptry protein 15(ROP15), and rhoptry protein 9 (ROP9), respectively (Table 1).

**Table 1:** *T. gondii* tachyzoite proteins identified by MALDI-TOF/MS and/or MS/MS

***SPOT NO.***	***Protein name***	***NCBI ID***	***PI***	***MW***	***Protein score***	***Protein Score C.I.%^a^***	***Sequence Coverage (%)^b^***	***No. of Matched peptides***	***Serum samples***
1	Enolase2	gi|672565998	5.67	48887	159	100	82.6	20	Sheep
2	ROP4	gi|6689341	6.28	42649	144	100	79.2	22	Sheep
3	GRA14	gi|1005150676	7.83	44671	82	99	90.8	12	Human
4	ROP15	gi|1005150012	8.20	34029	92	99	81.5	15	Human
5	ROP9	gi|1005151989	8.53	37963	170	100	87.0	20	Human

a C.I.% = the confidence interval for the Protein score.

b (Number of the matched residues/total number of residues in the entire sequence)×100%

The molecular weights of enolase 2, ROP4, GRA14, ROP15, and ROP9 were 48887 kDa, 42649 kDa, 44671kDa, 34029kDa and 37963 kDa, and also their isoelectric points were equal to 5.67, 6.28, 7.83, 8.2 and 8/53, respectively (Table 1).

## Discussion

We investigated immunoreactive profiles from tachyzoites of *T. gondii* using the combination of 2-DE, immunoblotting and mass spectrometric analysis. Proteins extracted from tachyzoites were resolved by 2-DE and subjected to western blot analysis probed with *T. gondii* positive sera from human and sheep.

Five antigenic spots that reacted with human and sheep anti-*T. gondii* sera were obtained by western blotting (from two gels). These spots were *T. gondii* immunoreactive proteins that matched in Swiss-Prot and NCBI nr database. Five antigenic proteins that we identified, include enolase2, ROP4 (from sheep serum), GRA14, ROP15 and ROP9 (from human serum).

During the last years, many studies have been done for identification of tachyzoites proteins, especially immunoreactive proteins. Many types of research have focused on the selection of immunoreactive and protective antigens, which could be candidate for vaccine against toxoplasmosis. Some of these proteins used in the combination of experimental vaccines are dense granule proteins GRA1 (29,30), GRA2 (31), GRA4 (32), GRA6 (33), rhoptry proteins ROP2 (34), ROP16 (35), ROP18 (36,37), micronemal proteins MIC3 (38,39), MIC6 (40), and surface antigen of tachyzoites SAG1 (41,42), SAG2 (43), SAG3 (44).

Enolases from several pathogens have been identified as important immunogenic proteins and protective antigens. This protein plays important roles in parasite metabolism, and it is likely a parasitic virulence factor. The enzymatic activity of *T. gondii* enolase2 was ion-dependent ant it was an important immunogenic protein of *T. gondii* (27).

*Toxoplasma gondii* rhoptry protein4 (ROP4), is a type I transmembrane protein that after secretion from the parasite during host cell division, associates with the vacuole membrane and becomes phosphorylated in the infected cell (45). Both rhoptry antigens ROP2 and ROP4 were induced humoral response (46). ROP4 was a very appropriate candidate for vaccine against chronic toxoplasmosis (47).

In the present study, enolase2 and ROP4 were identified as immunodominant and antigenic proteins of *T. gondii* tachyzoites, probed with *Toxoplasma* positive sera of sheep. Many rhoptry proteins have been showed to be key players in *T. gondii* invasion and virulence. Rhoptry neck proteins (RONs) including RONs2, 4, 5 and 8 have been invasion (16, 17); ROP16 activates the transcription factors STAT3 and STAT6 (18,19). At the time, RON12, ROP47, and ROP48 were not implicated in *Toxoplasma* virulence in mice (20). Some antigens of *T. gondii* including micronemal protein6 (MIC6) and rhoptry protein1 (ROP1) were identified only in the mice with cerebral tachyzoite growth (21). The recombination of GRA7, SAG1, and GRA8 antigens were proposed as recombinant proteins for use in IgG ELISA instead of *Toxoplasma* lysate antigens (TLAs) (22).

The list of dense granule proteins has grown during the past 20 yr since P23 (GRA1) was introduced as the first dense granule protein (48). Up to now fifteen proteins of 21–58 kDaa given the GRA designation have been characterized. GRA14 like many other dense granule proteins is a single hydrophobic α – *helix* predicted to encode membrane associated domains on secondary structure analysis. Indeed, this protein associates with membranous systems of PV such as MNN (membranous nanotubular network) or PVM (parasitophorous vacuolar membrane). Reduced in vitro growth rate under starvation condition is also observed for a cultured GRA14 KO (Knock-out) (49).

Chimeric *T. gondii* antigen, P35-MAG1 may be more useful than MIC1-ROP1 and MAG1-ROP1 in the preliminary detection of acute toxoplasmosis in humans (23). SAG1, GRA1 and GRA7 recombinant proteins with sensitivity of 100% and 91.1% for diagnosing the acute toxoplasmosis and the chronic infection respectively were proposed instead of TLAs in IgG ELISA test. GRA8, SAG2 and GRA6 recombinant proteins with 88.9% sensitivity and 100% specificity for diagnosis of chronic phase of toxoplasmosis were introduced (24). In 2008, MIC1ex2, MAG1 and MIC3 with 94.4% sensitivity and 100% specificity and in 2010, three recombinant proteins including GRA2, SAG1 and GRA5 with 93.1% sensitivity and 100% specificity, also three other recombinant proteins (ROP1, SAG1 and GRA5) with 94.2% sensitivity and 100% specificity were proposed to be used in IgG ELISA technique (25,26). Enolase2 could play important roles in metabolism, immunogenicity, and pathogenicity of *T. gondii* and that it may serve as a novel drug target and candidate vaccine against *Toxoplasma* infection (27).

GRA14 has a unique topology so that, C-terminal of this protein is located in the cytoplasm of host cell, and N-terminal is located in PV. Therefore, GRA14 can induce and stimulate the immune system of host (4).

Amongst the secreted proteins of *T. gondii,* rhoptry organelle proteins (ROPs) are essential for the parasite invasion. The contributions of fifteen ROPs (ROP10, ROP11, ROP15, etc.) were investigated for the infectivity of the high virulent type1 *T. gondii*. These fifteen ROPs including ROP15 might play different roles in life cycle of *T. gondii*. (50). ROP9 is a *T. gondii* rhoptry protein that is expressed by *T. gondii* isolates of all three intraspecies subgroups (51). ROP9 (spot 5) is a 38-kDaa protein, which has a homologue in *Plasmodium*. Indeed, both in *Toxoplasma* and *Plasmodium*, ROP9 (P36) is secreted from the rhoptry during invasion and is incorporated into the expanding PVM (52). In this study, three immunodominant and antigenic proteins of *T. gondii* were recognized by *Toxoplasma* positive sera of human: GRA14, ROP15, and ROP9.

## Conclusion

Enolase2 and ROP4 (in sheep) and GRA14, ROP15, and ROP9 (in human) were identified as immunodominant and antigenic proteins of *T. gondii*. Therefore, these five proteins have potential to be used as diagnostic antigens and markers of *T. gondii* infection in sheep and human.
